# Recent Advances in Fluorescence Resonance Energy Transfer (FRET) Biosensors for Exosomes

**DOI:** 10.3390/cimb47040235

**Published:** 2025-03-28

**Authors:** Feng Huang, Zhenyu Xie, Qianjiao Zhang, Shah Zada, Ruhan Lin, Yanmei Deng, Qifeng Liu, Huizhi Chen, Hui Zhou, Huilai Miao, Yubin Zhou

**Affiliations:** 1 Key Laboratory of Liver Injury Diagnosis and Repair, and Department of Hepatobiliary Surgery, The 2nd Affiliated Hospital of Guangdong Medical University, Zhanjiang 524001, China; huangfeng202202@163.com; 2 Dongguan Key Laboratory of Advanced Drug Delivery and Biosensing Research and Development, School of Pharmacy and Dongguan Innovation Institute, Guangdong Medical University, Dongguan 523808, China; 13342252715@163.com (Z.X.); qjzhang24@163.com (Q.Z.); shahzada@gdmu.edu.cn (S.Z.); 18320489200@163.com (R.L.); 15626892396@163.com (Y.D.); lqf980603@163.com (Q.L.); chenhuizhimail@126.com (H.C.); huizhou314@163.com (H.Z.)

**Keywords:** exosomes, biosensors, fluorescence, nanomaterials, fluorescence resonance energy transfer (FRET)

## Abstract

Cancer is a significant global health challenge, where early diagnosis is crucial for enhancing patient survival and mitigating the treatment burden on patients. Exosomes are extracellular vesicles released through the fusion of multivesicular bodies with cell membranes, carrying disease-associated information from donor cells. This makes exosomes a key biomarker in liquid biopsy analysis, particularly for early cancer detection. Developing cost-effective, straightforward, and sensitive exosome biosensing technologies is of significant practical importance. To date, a large number of fluorescence-based exosome biosensors have relied on the Fluorescence Resonance Energy Transfer (FRET) principle. This review introduces the basic background of the field and the principle of FRET-based exosome sensors, followed by a systematic summary of their progress categorized by different transduction elements or mechanisms. Finally, this work discusses the current challenges in the field and proposes potential solutions and future prospects, aiming to encourage and inspire the development of new approaches for advanced FRET exosome biosensors.

## 1. Introduction

Cancer has become the leading cause of mortality worldwide [1]. Early diagnosis plays a pivotal role in improving patient survival rates and alleviating the treatment burden on patients [2]. Liquid biopsy has emerged as a transformative approach in cancer early screening, diagnosis, treatment, and prognosis monitoring [3]. In contrast to traditional tissue biopsies, liquid biopsies offer several advantages, including being non-invasive, easy to sample, and having the ability to dynamically monitor cancer progression across different stages of development [4]. The primary biomarkers analyzed in liquid biopsy are circulating tumor cells (CTCs), circulating tumor DNA (ctDNA), and exosomes [5]. Among these, exosomes are particularly significant as they are abundant in biological fluids, provide comprehensive molecular information on donor cells, and their bilayer membrane structure effectively preserves molecular cargo for analysis [6,7,8]. As a result, exosome analysis has become a focus in the field of liquid biopsy, particularly for early cancer screening [9,10].

Exosomes are extracellular vesicles that are released when intracellular multivesicular bodies (MVBs) fuse with the cell membrane. They typically range in size from 30 to 150 nm and possess a lipid bilayer structure [11]. Specific exosome markers, known as Exosomes-Specific Markers (ESMs), include the Apoptosis-Linked Gene 2 interacting protein X (Alix), the tumor susceptibility gene 101 protein (TSG101), Heat-Shock Proteins (Hsp70, Hsp90), and Tetraspanins (CD9, CD63, and CD81) [12,13]. These markers play a crucial role in exosome isolation, purification, and identification. Exosomes carry a wide range of biomolecules derived from donor cells, including proteins, genetic material, such as non-coding RNAs (e.g., mRNA and microRNA), and lipids [14]. Notably, tumor-derived exosomes carry tumor-specific markers (Tumor-Specific Markers, TSMs), which are highly valuable for early tumor screening and diagnosis [15,16,17]. For example, the HER2 protein present in exosomes secreted by HER2-positive breast cancer cells serves as a valuable biomarker for diagnostic applications [18]. In addition, accumulating evidence has highlighted that cancer cell-derived exosomes play pivotal roles in cellular communication, modulating the tumor microenvironment and contributing to key oncogenic processes, including cancer cell proliferation, invasion, and angiogenesis [19]. Given the fluidity of biological body fluids, exosomes secreted by tumor cells can be detected in various biofluids, including blood, urine, tears, ascites, and saliva [11]. The lipid bilayer of exosomes encapsulates crucial bio-signaling molecules originating from donor cells, protecting them from enzymatic degradation and facilitating their stability and long-range transport in vivo [20]. This unique feature makes exosomes particularly advantageous for non-invasive liquid biopsies, holding significant potential as diagnostic tools for early cancer screening, monitoring tumor progression, and evaluating therapeutic responses without demanding invasive tissue biopsies. To facilitate the detection of exosomes, researchers have developed a variety of detection techniques. Conventional detection techniques include methods such as Western (WB) blotting, enzyme-linked immunosorbent assay (ELISA), flow cytometry, transmission electron microscopy (TEM), and nanoparticle tracking analysis (NTA). However, these methods often require specialized personnel and expensive equipment, presenting limitations in scalability and their ability to meet the routine demands of clinical applications [21,22]. In recent years, cost-effective, sensitive, and user-friendly biosensing technologies, such as fluorescence assays, electrochemical methods, Surface-Enhanced Raman Spectroscopy (SERS), surface plasmon resonance, and microfluidics, have been applied to different fields of biomedical applications, including the detection of exosomes [23,24]. Among these, fluorescence assays are particularly notable for their rapid sensitivity, rapid signal response, and strong adaptability, making them a popular strategy in the analysis of exosomes [25,26]. In addition, a large number of fluorescence-based exosome biosensors employ the Fluorescence Resonance Energy Transfer (FRET) principle. This review systematically summarizes the progress of FRET exosome biosensors classified with different transduction elements or mechanisms, including organic fluorescent dyes/quenching groups, carbon nanomaterials, Gold Nanoparticles (AuNPs), Upconversion Nanoparticles (UCNPs), Two-Dimensional materials (2D materials), Metal-Organic Frameworks (MOFs), and others. This work gives insights into the recent progress, current challenges, and prospects of FRET-based exosome biosensing and hopes to provide inspiration for the development of new strategies in the future.

## 2. Principle of FRET

**Figure 1:**
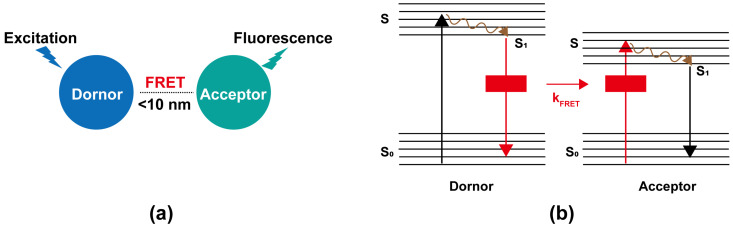
FRET principle schematic [30,31]. (**a**) When the distance of a donor and an acceptor with overlapping spectra is within 10 nm, the donor’s fluorescence is absorbed by the acceptor, which then emits fluorescence, initiating the FRET process. (**b**). Simplified Jablonski diagram. Upon excitation, the donor’s electron transits from the S_0_ to a S, undergoes vibrational relaxation to the S_1_, and transfers energy non-radiatively to the acceptor via FRET. This promotes the acceptor’s electron from the S_0_ to a S, followed by vibrational relaxation to the S_1_, ultimately emitting fluorescence as it returns to the S_0_.

The concept of FRET, also known as Förster Resonance Energy Transfer, was proposed by Theodor Förster in 1946 [27]. It is typically described as the process in which energy from a donor is transferred to an acceptor through a non-radiative (dipole–dipole) pathway when the donor and acceptor are in close proximity, typically 1–10 nm [28]. The efficiency of energy transfer is inversely proportional to the sixth power of the distance between the energy donor and acceptor, indicating its distance sensitivity [29]. This efficiency is also influenced by several factors, such as the degree of spectral overlap between the donor’s emission and the acceptor’s absorption, the relative orientation of the donor and acceptor molecules, the quantum yield of the donor, the fluorescence lifetime of the donor, and the absorption coefficient of the acceptor, among others [30]. This process is roughly demonstrated schematically in Figure 1. When the energy donor is excited and absorbs energy, its outer electron transitions from the ground state (S_0_) to a higher energy excited state (S) and undergoes vibrational relaxation to the first excited singlet state (S_1_). In the absence of an energy acceptor, the electron returns to the S_0_ by emitting fluorescence, and FRET does not occur. However, when an energy acceptor is present at a certain distance from the donor (i.e., less than 10 nm), the donor’s electron transfers energy to the acceptor electron via a non-radiative process. Subsequently, the acceptor electron transitions from the S_0_ to a S, undergoes vibrational relaxation to the S_1_, and eventually emits fluorescence as it returns to the S_0_. In this case, FRET occurs.

## 3. Progress of Exosome Biosensors Based on FRET

Since exosomes have emerged as prominent biomarkers for liquid biopsies, scientists have been actively exploring and developing various exosome detection methods. However, the precise detection of exosomes remains challenging due to their nanoscale size, low concentration in biological fluids, and suffering from interferences from the biological samples [32]. FRET-based biosensors have demonstrated significant potential in overcoming these challenges by offering high sensitivity, excellent specificity, distance-dependent signal modulation, rapid response times, simplicity, and homogeneous detection [33]. These unique properties enable FRET-based biosensors to enhance the accuracy and reliability of exosome analysis. FRET-based exosome biosensors generally employ aptamers, antibodies, or other specific binding molecules as biorecognition elements, with fluorescence serving as the signal output to facilitate the qualitative and quantitative detection of exosomes [34]. The fundamental design of FRET-based exosome biosensors typically involves an energy donor and an energy acceptor. In this review, we systematically categorize FRET biosensors for exosome detection based on the types of energy donors and acceptors applied, providing insights into their design principles and applications. Table 1 summarizes the biosensors developed for exosome detection using the FRET principle.

### 3.1. Based on Organic Fluorescent Dyes and Quenching Groups

Typical FRET-based exosome biosensors can be constructed with organic fluorescent dyes and quenching groups. Generally, these biosensors are designed in the form of a molecular beacon (MB) or an aptamer probe that relies on complementary strand hybridization. In these designs, the organic fluorescent dyes and quenching groups are either chemically modified onto the MB or attached to the aptamer and its complementary strand. In the absence of the target, MB forms a stable hairpin structure, while the aptamer binds to its complementary strand, bringing the organic fluorescent dye and quenching group into close proximity. This proximity suppresses fluorescence emission via FRET, keeping the signal in the “off” state. However, in the presence of the target, the MB hairpin is opened, and the aptamer dissociates from its complementary strand. This spatial arrangement induces fluorescence quenching via FRET, maintaining the system in an “off” state. In the presence of the target, the MB undergoes structural rearrangement, unfolding its hairpin conformation, and the aptamer dissociates from its complementary strand. This separation increases the donor-acceptor distance, effectively disrupting FRET and activating fluorescence, thereby switching the signal to the “on” state. In many cases, MBs incorporate aptamers due to their high specificity. An aptamer is a single-stranded DNA or RNA molecule obtained through the Systematic Evolution of Ligands by Exponential Enrichment (SELEX), a process that enables the selection of oligonucleotides with high affinity and specificity for their targets, earning them the designation of “artificial antibodies” [89,90]. Commonly used organic fluorescent dyes or quenching groups, such as fluorescein types (e.g., FITC, FAM), rhodamine types (e.g., rhodamine B, rhodamine 6G), cyanine dye types (e.g., Cy3, Cy5), QSY series (e.g., QSY 7, QSY 21), and Black Hole Quencher series (e.g., BHQ-1, BHQ-2, BHQ-3), provide various options when designing FRET exosome biosensors. They have advantages of low cost, high FRET efficiency, and being easy for covalent modification (i.e., onto exosome biorecognition elements, such as aptamers or antibodies). However, they face some issues, such as photobleaching, a relatively short fluorescence lifetime, and suffering interference from biological background fluorescence [91].

**Figure 2:**
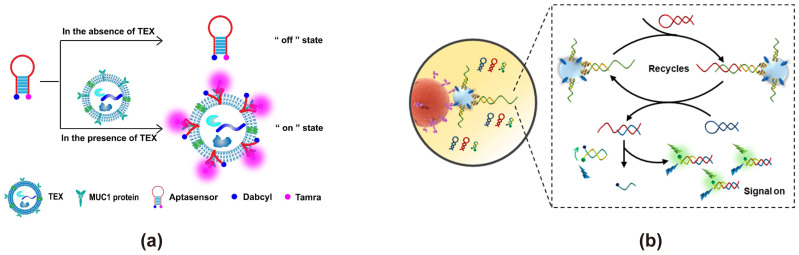
FRET exosome biosensors based on organic fluorescent dyes and quenching groups. (**a**) Biosensor design using a molecular beacon (MB) [36]. Upon the presence of tumor-derived exosomes, the aptasensor’s hairpin structure unfolds, restoring Tamra fluorescence and realizing detection. Images reproduced with permission. Copyright 2018, Elsevier. (**b**) Biosensor design using the complementary strand-based probe [35]. The bivalent cholesterol inserts into the exosomal membrane, triggering enzyme-free amplification that generates additional complementary H1 and H2 strands. These strands subsequently displace the RFQ probe strand, restoring FAM fluorescence and enabling exosome detection. Images reproduced with permission. Copyright 2021, American Chemical Society.

Junli Zhang developed a FRET-based fluorescent biosensor, targeting the breast cancer exosome marker MUC1 by conjugating the fluorophore Tamra as the energy donor and Dabcyl as the energy acceptor on the MB [36] (Figure 2a). In the absence of tumor-derived exosomes, the MB forms a stable hairpin structure, maintaining the signal in the “off” state due to efficient fluorescence quenching via FRET. However, upon binding to MUC1 on the exosome, the MB undergoes conformational changes, unfolding its hairpin structure, disrupting FRET, and restoring Tamra fluorescence. This FRET biosensor was able to successfully detect and differentiate exosomes derived from various cell lines, including MCF-7, MCF-7/ADR, A549, MGC-803, and Hs578Bst, as well as from the serum of breast cancer patients and healthy donors. The study demonstrated a significantly higher fluorescence signal in exosomes from breast cancer patient serum compared to healthy donors (*p* < 0.05), confirming its diagnostic potential. Another example is the complementary strand-based probe approach. Xiaokun Wang developed a FRET-based exosome biosensor by modifying the complementary strand with FAM as the energy donor and Dabcyl as the energy acceptor [35] (Figure 2b). In the presence of the target, exosome recognition triggers the dissociation of the complementary strand, disrupting FRET and restoring FAM fluorescence, thereby enabling exosome detection. The biosensor exhibited a detection range for exosomes spanning 5.5 × 10^3^ to 1.1 × 10^7^ particles/μL, with a limit of detection (LOD) of 1.29 × 10^3^ particles/μL. Additionally, the system demonstrated high analytical accuracy, achieving satisfactory recovery rates (92.25–106.8%) in the isolation and quantification of exosomes from various biological fluids.

### 3.2. Based on Carbon Nanomaterials

Given their exceptional physicochemical properties, such as high electrical conductivity, strong mechanical strength, a high surface area-to-volume ratio, and excellent biocompatibility, carbon nanomaterials are widely used for biosensors [92]. Among these, carbon nanotubes (CNTs) and graphene exhibit an extensive specific surface area and are rich in π-electrons, facilitating strong π-π stacking interactions with aptamer probes. When fluorescent probe-conjugated biosensors (i.e., aptamers) bind to these carbon nanomaterials, they serve as highly efficient energy acceptors, inducing fluorescence quenching [93]. In the presence of a target, the aptamer selectively interacts with it, leading to its dissociation from the carbon nanomaterial surface. This disruption of the FRET effect restores fluorescence, enabling both qualitative and quantitative target detection [94]. Carbon nanomaterials, including graphene oxide (GO), carbon nanotubes (CNTs), and carbon dots (CDs), are extensively utilized as key components in the design and fabrication of biosensors [95]. These biosensors have demonstrated efficient analysis capabilities for diverse analytes, including melamine [96], pathogens [97], pesticides [98], cholesterol [99], and exosomes [100].

**Figure 3:**
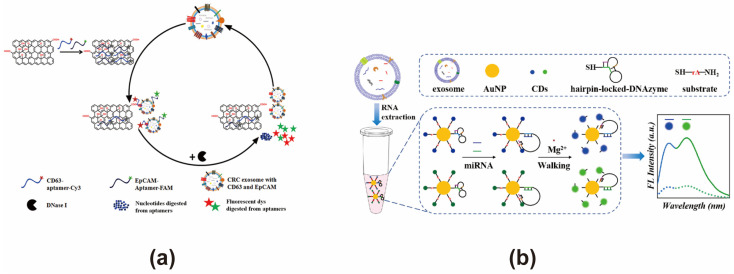
FRET exosome biosensors based on carbon nanomaterials. (**a**) Biosensor design using graphene oxide (GO) as the energy acceptor [52]. In the presence of exosomes, both CD63 and EpCAM aptamers dissociate from GO, leading to the restoration of Cy3 and FAM fluorescence. This process, combined with DNase I signal amplification, enables dual-target exosome detection. Images reproduced with permission. Copyright 2018, Elsevier B.V. All rights reserved. (**b**) Biosensor design using carbon dots (CD) as the energy donor [54]. In the presence of target miRNA, DNAzyme is activated, cleaving CD-labeled substrates and moving AuNPs along to restore fluorescence. Images reproduced with permission. Copyright 2022, American Chemical Society.

GO and CNTs are frequently employed as energy acceptors in the construction of FRET-based exosome biosensors. GO is a nanosheet obtained by oxidizing graphene sheets in a mixture of strong acids and oxidants, with the resulting oxygen-containing functional groups, such as hydroxyl and carboxyl groups, significantly enhancing its water solubility and providing the possibility for modification [101]. However, the presence of these oxygen-containing functional groups introduces a certain amount of sp^3^ hybridization into its sp^2^ structure, which may interfere with the π electron transfer between nucleic acid probes and GO, potentially affecting the FRET efficiency [102]. GO can quench the energy donors within an emission range of 200–800 nm, and, at the same time, it can also achieve a distance dependence that exceeds that of the traditional FRET quenching effect [103]. Its extremely large surface area makes it likely to adsorb multiple aptamer probes simultaneously, enabling the analysis of multiple target substances. Therefore, we believe that GO can be a superior energy acceptor for the simultaneous analysis of multiple targets on exosomes. However, this adsorption is prone to being disrupted by some non-specific substances other than targets, giving rise to high noise or background signals. CNTs are considered to be one-dimensional nanotubes with a nanoscale diameter, which can be formed by rolling up graphene sheets [104]. Similar to GO, CNTs can quench nucleic acid probes through π-π interactions. In contrast to GO, CNTs feature a fully sp^2^-hybridized structure, which may theoretically enhance their π-π interactions with nucleic acid probes, potentially leading to a highly efficient FRET quenching effect [105]. Moreover, due to its excellent near-infrared luminescence performance, it can act as an energy donor, providing an alternative solution for organic fluorescent dyes, the signals of which are in the UV-visible light range and are vulnerable to interference from background fluorescence [104]. When constructing FRET exosome biosensors, the water solubility of CNTs should be taken into account, which can be improved by modification design. Hui Wang developed a FRET biosensor using GO as an energy acceptor in combination with CY3/FAM-labeled CD63 and EpCAM aptamers, enhanced by DNaseI-assisted signal amplification. This biosensor achieved a detection limit of 2.1 × 10^4^ particles/μL for colorectal cancer (CRC)-derived exosomes and successfully distinguished between healthy individuals and CRC patients in 19 clinical serum samples. It also demonstrated potential for sensing exosomes from other tumor sources (Figure 3a) [52]. Zhiwei Sun developed a FRET biosensor utilizing MWCNTs@Au NCs and DSN-assisted signal amplification for the detection of exosome-associated miR-92a-3p from CRC. This biosensor achieved a detection range of 0.1–10 pM, with a detection limit of 31 fM under optimized conditions, and its accuracy was validated by measuring miR-92a-3p in clinical exosome samples [53]. Compared to conventional organic fluorescent dyes, carbon dots exhibit high quantum yields and can be excited in the near-infrared (NIR) region, making them attractive FRET fluorescence energy donors. Carbon dot-based approaches hold the potential to construct sensitive FRET-based exosome biosensors, enabling the detection of low-abundance biomarkers in exosomes from different sources. The NIR excitation range of carbon dots are beneficial for overcoming interference from biological background fluorescence. Xiao Zhang developed a DNAzyme Walker-type FRET exosome biosensor using carbon dots for the simultaneous detection of bladder cancer exosomal microRNAs (Figure 3b) [54]. In the presence of target miRNA, DNAzyme is activated, cleaving CD-labeled substrates and moving AuNPs along to restore fluorescence. This system achieved a detection limit at femtomolar levels and exhibited a linear detection range of 50 fM to 10 nM, while also enabling the simultaneous analysis of bladder cancer-related exosomal miR-133b and miR-135b in clinical serum samples.

### 3.3. Based on AuNPs

**Figure 4:**
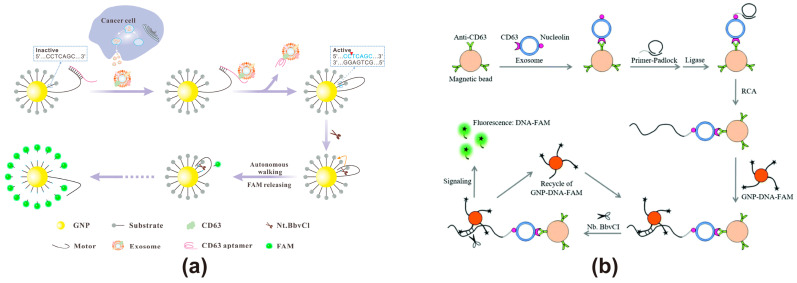
FRET exosome biosensors based on AuNPs. (**a**) Biosensor design using nanoflares with AuNPs [62]. Upon binding to the target exosome, the aptamer activates the DNA motor, initiating a stepwise process driven by a nuclease that cleaves the substrate strand, sequentially releasing and restoring FAM fluorescence. Images reproduced with permission. Copyright 2020, Elsevier B.V. All rights reserved. (**b**) Biosensor design using GNP-DNA-FAM conjugates for dual signal amplification [63]. Magnetic beads modified with CD63 antibodies capture exosomes, triggering rolling circle amplification and activating a nicking endonuclease, which sequentially cleaves the substrate strand, leading to the stepwise release and restoration of FAM fluorescence. Images reproduced with permission. Copyright 2018, Royal Society of Chemistry (RSC).

AuNPs exhibit unique advantages and significant application potential as FRET energy acceptors in the field of biosensing. Due to their tunable optical properties, including adjustments in particle size and morphology, excellent stability, easily modifiable surfaces, significant molar extinction coefficients, and broad absorption bands in the visible spectrum, along with their high biocompatibility, they are promising candidates for applications in biosensing and analytical chemistry [106]. Due to the absence of a definite dipole moment, AuNPs possess a quenching ability over an extremely long distance of 60–100 nm, which is 10 times the distance range of the traditional FRET effect [103]. When an extremely long-distance quenching is required, AuNPs can be a good choice as an energy acceptor. However, the relatively high cost of the core element Au may be a factor that needs to be taken into account. AuNPs typically range in size from 1 to 100 nm. When the emission spectrum of an energy donor overlaps with the surface plasmon resonance (SPR) absorption spectrum of AuNPs, fluorescence quenching occurs. For instance, AuNPs with a diameter of 13 nm appear red and exhibit an SPR absorption peak at 520 nm [107]. As the particle size increases, the SPR absorption peak undergoes a red shift to longer wavelengths, while the absorption spectrum broadens. Therefore, AuNPs of different sizes can be utilized to quench energy donors emitting at various wavelengths, enabling precise modulation of the FRET process. Additionally, AuNPs can regulate the grafting rate of fluorescent probes by adjusting their particle size. Studies have shown that AuNPs of larger size possess lower surface curvature, leading to greater repulsive forces between fluorescent probes, which in turn reduces the modification density [108]. Furthermore, gold nanoclusters (AuNCs), which consist of relatively small AuNPs (typically <2.2 nm), can serve as excellent energy donors [109]. Due to their relatively long emission lifetimes, AuNCs can be utilized for the construction of biosensors with extended detection durations. Currently, AuNCs are commonly used in constructing molecular probes for the in vivo imaging of exosomes [110]. However, reports on employing AuNCs as energy donors to construct FRET exosome biosensors are scarce. Yanyan Yu developed nanoflares for exosome detection using AuNPs as a core sensing platform, as shown in Figure 4a [62]. The nanoflares, incorporating a 3D DNA motor, specifically recognize tumor-derived exosomes through aptamer-based molecular recognition. Upon aptamer binding to the target exosome, the DNA motor is activated, initiating a stepwise process driven by nicking endonucleases that cleave the substrate chain, sequentially releasing fluorescent molecules. This mechanism facilitates fluorescence-based signal amplification, effectively compensating for RNA base instability. The method exhibits a broad dynamic range spanning five orders of magnitude for exosome detection, achieving a detection limit as low as 8.2 particles/μL, with excellent selectivity for exosomes from different tumor sources and robust performance in complex biological samples. Lin Huang developed a fluorescence biosensing platform using GNP-DNA-FAM conjugates to construct nanoflares, enabling the ultrasensitive detection of exosomes derived from leukemia cells through dual signal amplification (Figure 4b) [63]. The method captures leukemia-derived exosomes using anti-CD63 antibody-modified magnetic beads (MB-CD63), then triggers rolling circle amplification (RCA) with AS1411-containing DNA primers. This generates repetitive sequences that hybridize with GNP-DNA-FAM conjugates, followed by target recovery assisted by the nicking endonuclease (Nb·BbvCI), which releases FAM from the GNP-DNA-FAM conjugates, leading to the continuous accumulation of fluorescence signals. This dual signal amplification strategy exhibits strong resistance to serum interference and achieves a detection limit as low as 1 × 10^2^ particles/μL, demonstrating its potential for highly sensitive exosome detection in clinical samples.

### 3.4. Based on UCNPs

**Figure 5:**
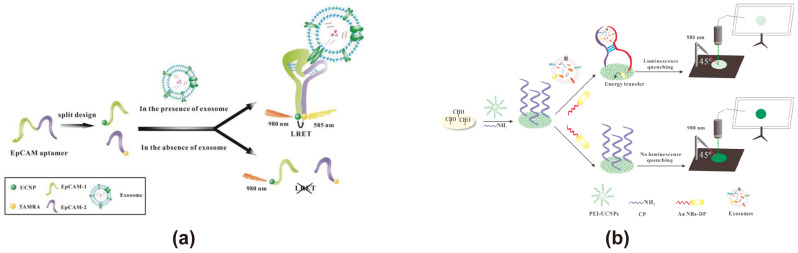
FRET exosome biosensors based on UCNPs. (**a**) Biosensor design using NaYF4: Yb, Er UCNPs, and TAMRA for exosome detection [71]. EpCAM aptamer 1 and EpCAM aptamer 2 simultaneously recognize exosomes, restricting NaYF4: Yb, Er UCNPs, and TAMRA within an optimal FRET distance. Upon excitation at 980 nm, NaYF4: Yb, Er, and UCNPs transfer energy to TAMRA via FRET, inducing fluorescence emission at 585 nm. Images reproduced with permission. Copyright 2019, Elsevier B.V. All rights reserved. (**b**) Biosensor design utilizing UCNPs and Au NRs with nucleic acid aptamer technology [72]. The CD63 aptamer is divided into two fragments, functionalized with UCNPs and gold nanorods, and immobilized on a paper-based sensing platform. In the presence of exosomes, the aptamer fragments bring UCNPs and Au NRs into proximity, triggering UCNP quenching for exosome detection. Images reproduced with permission. Copyright 2018, Elsevier B.V. All rights reserved.

UCNPs exhibit a distinct fluorescence emission mode from traditional fluorescent spheres by absorbing two or more low-energy photons at longer wavelengths and emitting higher-energy photons at shorter wavelengths (anti-Stokes shift luminescence). This process occurs via three mechanisms, such as Excited-State Absorption (ESA), Energy Transfer Upconversion (ETU), and Photon Avalanche (PA) [111]. UCNPs consist of a host matrix, an activator, and a sensitizer. The host matrix is designed for high tolerance, stability, and efficient photon migration; the activator exhibits a long lifetime in intermediate states; and the sensitizer features a large absorption cross-section, matching energy levels, and excited states in the NIR spectrum [112]. These materials, which absorb energy in the near-infrared (NIR) region and emit fluorescence in the visible light range, offer significant advantages in biosensing applications. By minimizing donor-acceptor co-excitation, UCNPs effectively prevent photodamage to biomolecules such as DNA, significantly reducing background fluorescence interference and lowering the risk of false-positive signals [113]. When the biological background interference in the UV-visible light range is strong, UCNPs can be used as the energy donor. Although near-infrared light has strong tissue penetration ability, enabling UCNPs to achieve in vivo imaging at relatively deep levels, this low-energy excitation leads to a low photoluminescence quantum yield. In this situation, using UCNPs as the energy donor of the FRET exosome biosensor may affect its sensitivity in detecting exosomes. Moreover, traditional UCNPs are usually excited at 980 nm [114]. However, water molecules can absorb the light at 980 nm and convert it into heat energy, thereby heating the sample, which may be detrimental to the exosome samples being detected. Using Nd^3+^ ions as a sensitizer may, as much as possible, solve this problem. In recent years, pertinent research efforts have been devoted to enhancing the photoluminescence quantum yield of UCNPs and thereby augmenting their photoluminescence capabilities via means such as nanostructure engineering, plasmonic regulation, dye sensitization, and modification of the core-shell architecture [115]. Overall, UCNPs are capable of acting as an outstanding energy donor for FRET-based exosome biosensors. In FRET-based exosome biosensors, UCNPs with unique fluorescence properties are typically used as fluorescent energy donors. Yonghao Wang proposed a strategy based on the luminescence resonance energy transfer (LRET) process between NaYF4: Yb, Er UCNPs and tetramethylrhodamine (TAMRA) for the highly sensitive detection of exosomes derived from breast cancer cells, with EpCAM as the target molecule (Figure 5a) [71]. In this process, the EpCAM aptamer is cleaved into two DNA strands (EpCAM-1 and EpCAM-2), each modified with UCNPs and TAMRA, respectively. Upon recognition and binding to the EpCAM protein on exosomes, a hairpin structure forms, bringing UCNPs and TAMRA within 10 nm, enabling LRET. When excited with 980 nm near-infrared light, TAMRA emits yellow fluorescence at 585 nm, allowing quantitative analysis of exosomes based on changes in fluorescence intensity. Xiaosong Chen designed an analytical strip for exosome detection based on LRET and nucleic acid aptamer technology (Figure 5b) [72]. In this biosensor, the CD63 DNA aptamer is split into two fragments, functionalized with UCNPs and gold nanorods (Au NRs), and immobilized on a filter paper-based sensing platform. Upon binding to the CD63 protein on exosomes, the aptamer fragments draw the UCNPs and Au NRs into close proximity, triggering the LRET process. This design enables exosome detection with a linear range of 1.0 × 10^4^ to 1.0 × 10^8^ particles/μL and a detection limit of 1.1 × 10^3^ particles/μL.

### 3.5. Based on 2D Materials

**Figure 6:**
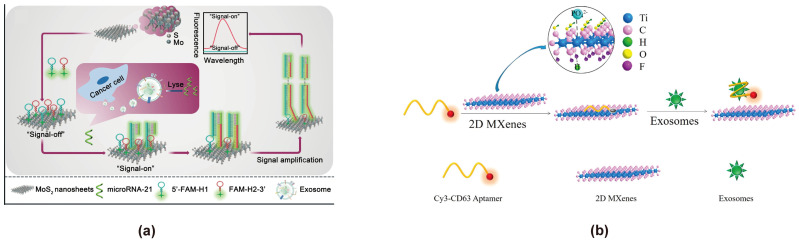
FRET exosome biosensors based on 2D materials. (**a**) Biosensor design using MoS_2_ nanosheets as the energy acceptor [74]. In the presence of tumor exosome-derived microRNA-21, the HCR process is activated, generating FAM-labeled amplification products that emit strong fluorescence at 518 nm, enabling highly sensitive detection. Images reproduced with permission. Copyright 2021, Springer Nature. (**b**) Biosensor design using Ti_3_C_2_ MXenes as the energy acceptor [75]. In the presence of exosomes, the CD63 aptamer detaches from Ti_3_C_2_ MXenes, leading to the restoration of Cy3 fluorescence. Images reproduced with permission. Copyright 2018, American Chemical Society.

In 2004, Andre Geim and Konstantin Novoselov successfully exfoliated a single layer of graphene from bulk graphite, sparking the exploration of 2D materials with unique electronic, optical, and physicochemical properties. These properties include efficient charge transfer, large surface area, transparency, biocompatibility, flexible structures, and strong interface interactions with biological entities such as cells, DNA, and protein. On the other hand, recent development of polymers has extended their applications in sensing applications, which is beneficial for their incorporation with 2D materials [116,117,118,119]. Due to their unique properties, 2D materials have been progressively applied in the development of electrochemical and optical biosensors for the analysis of nucleic acids, proteins, small molecules, and other analytes in recent years [120]. In the context of FRET-based biosensors for exosome detection, 2D materials serve as highly efficient energy acceptors due to their high surface area, excellent biocompatibility, and strong adsorption capabilities for aptamers, exhibiting strong fluorescence-quenching. These features make them ideal platforms for enhancing sensitivity and specificity in exosome detection. Among them, 2D materials such as MoS_2_, Ti_3_C_2_ MXenes, and MnO_2_ are commonly used in the construction of FRET exosome biosensors. They are generally nanosheets composed of transition metals and non-metallic elements. There are differences in the adsorption modes and interaction force magnitudes of aptamer probes among different 2D materials. MoS_2_ exhibits strong adsorption of aptamer probes mainly through van der Waals forces [121]. Ti_3_C_2_ MXenes primarily exert strong chemisorption, while MnO_2_ achieves strong adsorption of aptamer probes through interaction with their phosphate backbones [122,123]. The forces by which Ti_3_C_2_ MXenes and MnO_2_ adsorb aptamer probes may both be stronger than the π-π interaction forces by which GO adsorbs aptamer probes. The different adsorption capacities of energy acceptors for aptamer probes may lead to different quenching intensities of energy donors, thereby affecting the sensitivity of FRET-based exosome biosensors for exosome detection. Jinlan Wei reported a simple label-free nanoplatform for the quantitative detection of exosome-derived microRNA-21 from lung cancer plasma, using molybdenum disulfide (MoS_2_) nanosheets as fluorescence quenching agents (signal off) and hybridization chain reaction (HCR)-assisted signal amplification (signal on) (Figure 6a) [74]. In this approach, FAM-labeled hybrid probes (FAM-H1 and FAM-H2) complementary to the microRNA-21 sequence are designed. MoS_2_ nanosheets adsorb the hybrid probes, effectively quenching their fluorescence and minimizing background signals. Upon recognition of microRNA-21, the HCR process is activated, generating FAM-labeled amplification products that emit strong fluorescence at 518 nm, enabling highly sensitive detection. This system achieves a detection limit of 6 pmol/L with a broad linear detection range of 0.01–25 nmol/L, demonstrating its potential for clinical diagnostics. Qiuxia Zhang developed a novel FRET-based nanoprobe for quantitative exosome detection, utilizing a Cy3-labeled CD63 aptamer in combination with Ti_3_C_2_ MXenes nanocomposites as self-referenced ratio sensors (Figure 6b) [75]. The process involves the selective adsorption of Cy3-CD63 aptamer onto Ti_3_C_2_ MXenes through hydrogen bonding and metal coordination, resulting in fluorescence quenching via FRET. Upon addition of exosomes, fluorescence is restored as the aptamer is released. This method achieves a detection limit of 1.4 × 10^3^ mL^−1^, over 1000 times lower than conventional ELISA, highlighting its potential for high-performance exosome analysis. Juan Chen constructed a FRET exosome biosensor by using MnO_2_ as an energy acceptor to quench fluorescent microspheres, enabling accurate and portable detection of tumor exosomes through a dual-mode lateral flow chromatography assay [88]. This provides a promising solution for the point-of-care testing (POCT) of tumor exosomes.

### 3.6. Based on MOFs

**Figure 7:**
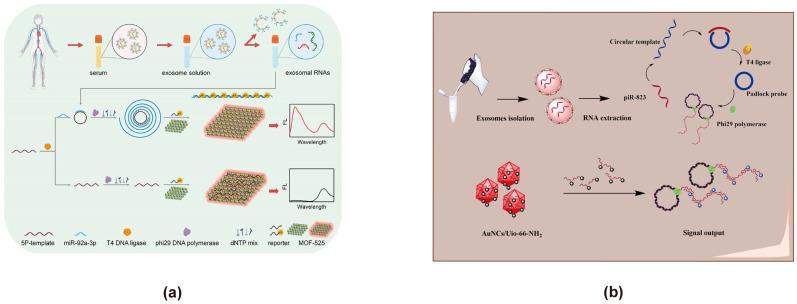
FRET exosome biosensors based on MOFs. (**a**) Biosensor design using self-fluorescent MOF-525 with an RCA strategy for miR-92a-3p detection [80]. In the presence of exosome-derived RNAs, RCA is triggered, facilitating the hybridization of RNAs with the reporter DNA to form double-stranded structures, preventing their adsorption onto MOF-525. As a result, the fluorescence of the reporter remains unquenched. The exosome-derived RNAs are detected through ratiometric fluorescence analysis by comparing the fluorescence of the reporter group with the intrinsic fluorescence of MOF-525. Images are reproduced with permission. Copyright 2024, Elsevier B.V. All rights reserved. (**b**) Detection of Exosome-Derived piRNA-823 Using Au NCs and UiO-66-NH_2_ MOF [81]. Exosomal RNA undergoes sequential ligase-mediated ligation and polymerase amplification. The reporter probe hybridizes with the amplified product, leading to the desorption of Au NCs from UiO-66-NH_2_ MOFs, generating a ratiometric signal output for the quantitative detection of exosome-derived piR-823. Images reproduced with permission. Copyright 2024, Elsevier B.V. All rights reserved.

MOFs are crystalline porous materials formed through the self-assembly of inorganic metal nodes and organic ligands. They are characterized by highly flexible framework structures, complex topologies, and large surface areas. Nano-scale MOFs (NMOFs) offer additional advantages over bulk MOFs, including faster signal responses, enhanced monodispersity, improved stability, and broader liquid-phase applications [124]. In FRET sensor design, MOFs are often employed as carriers for energy donors or acceptors. Their easily tunable porosity and large specific surface area significantly enhance the loading capacity for both energy donors and acceptors. Moreover, MOFs facilitate the uniform distribution of energy donors within their pores, thereby preventing aggregation-induced photobleaching. This not only improves the stability of the energy donors but also boosts their emission intensity, which in turn enhances the sensitivity of exosome detection. Additionally, MOFs enable the incorporation of various energy donors, such as organic fluorophores, quantum dots, carbon dots, and UCNPs, which further improves donor performance. They can also precisely regulate the distance between energy donors and acceptors, thereby increasing the efficiency of FRET energy transfer. An additional advantage is the improved stability of both the energy donors and acceptors, resulting in a long-lasting, stable signal that allows for prolonged exosome monitoring. Therefore, we believe that integrating MOFs into the design of FRET-based exosome biosensors may greatly optimize sensor performance. MOF-525 exhibits intrinsic photoluminescence and can serve as an energy donor by itself. Zhiwei Sun developed a ratio fluorescence exosome FRET biosensor based on self-fluorescent MOF-525 and a target-triggered RCA strategy for detecting CRC-related miR-92a-3p extracted from exosomes (Figure 7a) [80]. MOF-525, which serves a dual function as both a quenching group and a fluorescence reference, enables the detection of miR-92a-3p through a RCA strategy. In this system, the fluorescence intensity ratio (Δreporter/MOF-525) is directly proportional to miR-92a-3p concentration within the range of 0.1–10 pM, allowing for precise quantification and effective discrimination from mismatched RNA sequences. Yao Tong developed a biosensor for detecting exosomal piRNA-823 using a MOF with the UiO-66 prototype structure, which supports Au NCs (Figure 7b) [81]. In this CRC exosome detection process, piRNA-823 undergoes ligase and polymerase amplification, with the reporter gene binding to the amplification product and preventing adsorption by the Au NCs/MOF. This mechanism generates a ratio signal for quantification, demonstrating a wide linear range of 0.04–4 pM and a detection limit of 10.2 fM for piRNA-823.

### 3.7. Others

**Figure 8:**
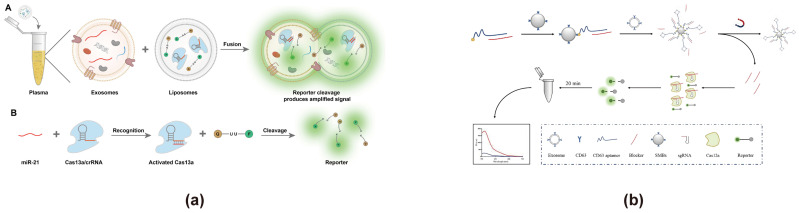
Exosome biosensors based on CRISPR technology. (**a**) Biosensor design using liposome-mediated CRISPR/Cas13a for miR-21 detection in exosomes [83]. (**A**) Exosomes fuse with liposomes containing Cas13a/crRNA and the reporter. (**B**) Upon binding with miR-21 in the exosomes, the CRISPR system is activated, cleaving the reporter and separating the energy donor and acceptor, which disrupts the FRET effect and generates a fluorescence signal for detection. Images are reproduced with permission. Copyright 2023, American Chemical Society. (**b**) Biosensor design using CRISPR/Cas12a and the Apt63-SMB complex for exosome detection [82]. When exosomes are present, the blocker dissociates from the CD63 aptamer and activates CRISPR/Cas12a to cleave the reporter, disrupting the FRET effect and restoring fluorescence. Images reproduced with permission. Copyright 2024, Springer Nature.

In addition, there have been some emergent strategies for FRET-based exosome biosensor design. For example, biosensors have been reported that could achieve signal output by disrupting FRET through CRISPR technology. CRISPR is a cutting-edge RNA detection technology consisting of three main stages: during the adaptation stage, the CRISPR locus acquires new spacer sequences; in the expression stage, the Cas gene is expressed, and the CRISPR sequence is transcribed into precursor crRNA, which is processed into mature crRNA with the assistance of Cas proteins and other cofactors; and finally, in the interference stage, crRNA, together with Cas proteins, identifies and cleaves the target viral DNA [125,126]. CRISPR technology has been involved in the construction of exosome sensors [127]. For example, CRISPR technology is frequently used to cleave reporter molecules, facilitating the detection of RNA biomarkers derived from exosomes. The reporter group is typically designed based on conventional FRET principles for fluorescence quenching and activation. Junli Zhang utilized liposome-mediated transfection of CRISPR/Cas13a to efficiently detect exosomal miR-21 in plasma (Figure 8a) [83]. In this approach, exosomes fuse with liposomes containing the CRISPR system, crRNA, and a reporter probe. Upon recognition of miR-21 in the exosomes, the CRISPR system is activated, leading to the cleaving of the reporter probe. This disrupts FRET by separating the energy donor and acceptor, generating a fluorescence signal for detection. This approach achieved a linear detection range spanning four orders of magnitude (10^4^–10^8^ particles/mL), with a detection limit as low as 1.2 × 10^3^ particles/mL. In addition to RNA detection, researchers have explored CRISPR technology for exosome surface protein analysis. Xianxian Zhao developed a CRISPR/Cas12a-based biosensor for the rapid and sensitive detection of exosomes via CD63 surface protein recognition (Figure 8b) [82]. In this method, the Apt63-SMB complex captures exosomes, inducing a conformational change in the CD63 aptamer that releases the blocker. The released blocker is subsequently recognized by the CRISPR/Cas12a system, which triggers trans-cleavage of the reporter group, producing a fluorescent signal. This method demonstrated a detection range of 3 × 10^3^–6 × 10^7^ particles/μL and effectively distinguished exosomes from clinical samples of healthy individuals and lung cancer patients. Apart from the aforementioned methods, some new approaches were recently reported. For instance, Reshma Bano constructed an exosome biosensor by exciting Enhanced Cyan Fluorescent Protein (ECFP) at 420 nm and making Venus emit a fluorescent signal at 540 nm through FRET, which involved two different fluorophores for energy transfer [86]. This enabled a simple, rapid, sensitive, and non-invasive monitoring of exosomes from A549 cells. In order to further improve the sensitivity and accuracy of exosome detection, scientists have also combined FRET with new technologies, such as microfluidics, signal amplification, and dual-mode output strategies. Jaewoo Lim combined FRET with microfluidic technology and signal amplification technology to detect exosomes, achieving a sensitive detection of the breast cancer biomarker ERBB2 [87]. Its detection limit is 58.3 fM, and it enables the trace detection of samples with a volume of ≤100 μL.

## 4. Challenges and Prospects

The development of innovative exosome biosensors plays a crucial role in advancing non-invasive diagnostics and disease monitoring. FRET has significant application value in exosome detection. We systematically summarized the FRET-based exosome biosensors designed with various elements, including organic fluorescent dyes/quenching groups, carbon nanomaterials, AuNPs, UCNPs, 2D materials, MOFs, and so on. Here are the potential challenges that may arise in the design of FRET-based exosome biosensors and the corresponding strategies to address them:

The reported FRET biosensors have made great efforts in the detection of exosomes. However, different approaches or strategies may face different main technical challenges. Energy donors (such as organic fluorescent dyes and carbon dots) in the relatively short wavelength range (like the UV-visible range) have demonstrated broad applications, but they may face technical challenges, including photobleaching and interference from background fluorescence. In contrast, energy donors (such as UCNPs) in the relatively long wavelength range (such as NIR) may solve these interferences but may suffer from low-energy excitation, leading to weak luminescence, as well as the heat generation effect caused by water absorption at 980 nm. To overcome these challenges, the introduction of excellent carrier materials, such as MOFs, offers a potential solution. MOFs possess tunable porosity and a large specific surface area, which can effectively regulate the distance between energy donors or between energy donors and acceptors, while also enhancing the stability of energy donors to improve their luminescence intensity. Additionally, the development of optimized NIR organic fluorescent dyes and UCNPs with higher luminescence intensity could further enhance sensing performance. On the other hand, quenching materials, such as GO, MoS_2_, and MnO_2_, may primarily face the challenge of nonspecific desorption. The use of more specific biorecognition elements may effectively mitigate interference caused by nonspecific adsorption. Additionally, the current approaches have shared challenges in detecting exosomes from different sources, i.e., the complexity of biological samples and the low concentration of exosomes. Biological samples contain not only target analytes (exosomes) but also various interfering substances, such as various proteins, nucleic acids, and ions, which may play multiple roles influencing the sensing performance. These interfering substances may increase background signals through intrinsic fluorescence, quench the energy donors to weaken output signals, or cause nonspecific binding with biorecognition elements, thereby affecting detection accuracy. Therefore, the effective pretreatment of exosomes from different sources is crucial before detection. For instance, high-efficiency sample separation techniques, such as immunoaffinity columns or microfluidics, can maximize the removal of impurities and improve detection specificity and sensitivity. The nanoscale size and low concentration of exosomes further complicate detection, but signal amplification strategies like RCA and HCR have been employed to enhance sensitivity, as exemplified by the works of Zhiwei Sun and Jinlan Wei [74,80]. CRISPR technology has also been integrated to achieve ultra-sensitive detection, as demonstrated by Junli Zhang’s work on exosomal miR-21 detection [83].

Through a comprehensive review and discussion of the current FRET biosensor for exosome detection, some potential research directions and application prospects are proposed. In the future, FRET-based exosome biosensors are anticipated to evolve toward the more precise, rapid, and convenient direct analysis of biological fluids, eliminating the need for extensive and cumbersome preprocessing steps. The integration of high-performance energy donors, energy acceptors, and MOFs is expected to significantly contribute to these advancements. Moreover, coupling these sensors with emerging technologies, such as microfluidic systems, could enable high-throughput, accurate, and user-friendly exosome detection. These miniaturized platforms offer notable advantages, including reduced sample consumption, the elimination of preprocessing, shortened detection times, and enhanced portability, making them strong candidates for POCT. However, when implementing FRET-based exosome biosensors for POCT, it is crucial to address the challenges of light source dependence and the miniaturization of both the light source and fluorescence signal processing instruments. Furthermore, artificial intelligence (AI)-incorporated sensors are a promising direction in which to move forward, and the incorporation of AI into FRET-based exosome biosensors enables the automated analysis of complex biological signals, facilitating real-time disease diagnosis and predictive analytics [128]. In the design and performance optimization of FRET-based exosome biosensors, AI’s robust capabilities in data analysis, logical reasoning, and molecular simulation can be harnessed to guide the selection of biorecognition elements, energy donors, and energy acceptors. For example, molecular simulations can be performed in advance to evaluate the binding affinities and release kinetics of biorecognition elements, such as aptamers or antibodies, with both energy donors and exosome targets. Additionally, the optimal energy donors and acceptors can be selected based on their spectral overlap, while appropriate FRET distances can be used to determine the optimal modification sites on the aptamers or antibodies. In the realm of data processing, AI can facilitate the automated acquisition, organization, and analysis of data, thereby generating professional exosome detection reports. Moreover, by leveraging machine learning, AI continually enhances its analytical capabilities to predict the correlation between exosome detection outcomes and disease states. As research continues to push the boundaries of biosensor technology, the integration of FRET with next-generation nanomaterials, AI, and microfluidic platforms will pave the way for more efficient, accurate, and accessible exosome-based diagnostics, ultimately contributing to the advancement of precision medicine. This article aims to provide a reference for the development of more advanced FRET-based exosome biosensors with superior performance.

## Figures and Tables

**Table 1:** Biosensors developed for exosome detection using the FRET principle.

Category	Donor/Acceptor	Exosome Target	References
Based on organic fluorescent dyes and quenching groups	FAM/Dabcyl	CD63	[35]
	TAMRA/Dabcyl	MUC1	[36]
	FAM/TAMRA	CD63, EGFR, EpCAM	[37]
	FAM/BHQ1	miR-141	[38]
	CY3/CY5	miR-200c-3p, miR-222-3p, miR-375-3p	[39]
	FAM/BHQ1	miR-21	[40]
	CY3/CY5	PD-L1	[41]
	Fluorescein/PPDox	CD63	[42]
	FAM, Cy3, Cy5/BHQ1, BHQ2	miR-21, miR-27a, miR-375	[43]
	FAM/BHQ1	miRNA-21	[44]
	FAM/BHQ1	miRNA-21	[45]
	Cy3/BHQ2	CD63	[46]
	FAM/BHQ1	miRNA-27a	[47]
	FITC/BHQ1	PSMA	[48]
	TAMRA/Dabcyl	PSMA	[49]
Based on carbon nanomaterials	FAM/GO	CD63, MUC1	[50]
	FITC/GO	CD63	[51]
	CY3, FAM/GO	CD63, EpCAM	[52]
	Atto-425/MWCNTs@Au NCs	miR-92a-3p	[53]
	CDs/AuNPs	miR-133b, miR-135b	[54]
	CDs/DSA	miRNA-21	[55]
	FAM/GO	CD63	[56]
	TPE-TA/GO	PSMA	[57]
	FAM/GO	PSMA	[58]
	FAM-GO	Aβ42	[59]
	FAM/GO	miR-193a	[60]
	FAM/CCM	Mir210	[61]
Based on AuNPs	FAM/AuNPs	CD63	[62]
	FAM/AuNPs	CD63	[63]
	QD/AuNPs	EpCAM	[64]
	Cy5/Au nanoflare	MicroRNA-1246	[65]
	Atto-425/AuNPs	miR-92a-3p	[66]
	AuNCs/PDANS	Aβ42, CD63	[67]
	FAM/AuNPs	CD63	[68]
	FAM, HEX, Cy5/Au nanoflare	miR-21, 122, 375	[69]
	FAM, Cy5/AuNPs	PD-L1, ExomiR-21	[70]
Based onUCNPs	UCNPs/TAMRA	EpCAM	[71]
	UCNPs/AuNRs	CD63	[72]
	NaYF4: Tb/BHQ1	EpCAM	[73]
Based on 2D materials	FAM/MoS_2_	microRNA-21	[74]
	Cy3/Ti_3_C_2_ Mxenes	CD63	[75]
	PE/MoS_2_-MWCNT	CD63	[76]
	FAM/MoS_2_	miRNA-21	[77]
	FAM/Ti_3_C_2_	CD63	[78]
	Alexa Fluor 647/BP@Mn^2+^	miR-21	[79]
Based on MOFs	FAM/MOF-525	miR-92a-3p	[80]
	Au NCs/UiO-66-NH	piR-823	[81]
Others	Cy3/BHQ	CD63	[82]
	Cy5	miR-21	[83]
	FAM	miRNA-21	[84]
	FAM/BHQ1	miRNA-21	[85]
	ECFPs/Venus	EGFR	[86]
		ERBB2	[87]
	FMs/MnO_2_	MUC1	[88]
